# Public health surveillance and information technology.

**DOI:** 10.3201/eid0403.980333

**Published:** 1998

**Authors:** R W Pinner

**Affiliations:** Centers for Disease Control and Prevention, Atlanta, Georgia, USA.


### Public Health Surveillance and Information Technology

**Robert W. Pinner**
Centers for Disease Control and Prevention, Atlanta, Georgia, USA

### Applying Modern Information Technology to Reporting for Public Health—the Role of Standards

Clement McDonald, Indiana University School of Medicine, discussed the role of standards in the application of modern information technology to public health reporting. He pointed to the rich data sources stored electronically in clinical laboratories, pathology and cytology reporting systems, pharmacies, and hospitals, and emphasized the trend toward increasing automation.

Interest and demand for electronic delivery of data come from many interested parties—3rd party payers, researchers, physicians, and public health officials. However, substantial barriers to smooth electronic flow of this information include the storage of data in isolated areas, varying internal structures among information systems, and considerable variation in codes (e.g., for laboratory tests and results). Overcoming these barriers requires defining, adopting, and implementing standards for messages, codes, identification (e.g., persons, providers, places), and security.

### Messages

Health Level Seven (HL7) is a message standard that defines messages for laboratory and other clinical results, immunization reporting, drug usages, patient registration, and clinical trials. HL7 provides standards for the structure and organization of clinical messages, defining data types, and structure of the "records" in the message. A 1997 Healthcare Information Management System Societies/Hewlett-Packard Leadership Survey found that HL7 was the most important health informatics standard. HL7 is an American National Standards Institute (ANSI)–approved clinical message standard used widely in the United States and internationally. Additional information can be found at the HL7 Internet web site: http://www.mcis.duke.edu/standards/HL7/hl7.htm.

### Codes

Code standards include Logical Observations Identifiers Names and Codes (LOINC), a code standard that identifies clinical questions, variables, and reports; Systematized Nomenclature of Medicine (SNOMED), which identifies procedures and possible answers to these questions, such as test results; Current Procedural Terminology, Version 4 (CPT4), which identifies procedures; and the National Library of Medicine's Unified Medical Language (UMLS), a metathesaurus of most code systems.

LOINC comprises a database of 15,000 variables with synonyms and cross-mappings and covers a wide range of laboratory and clinical subject areas (e.g., blood bank, chemistry, hematology, microbiology, vital signs, body measurements, obstetric ultrasound, and electrocardiograms). LOINC's formal naming structure has six parts: component (analyte); property measured; time aspect; system (specimen, organ), precision, method. LOINC is being adopted by several large reference laboratories, and it has been incorporated into UMLS. Additional information about LOINC can be found at http://www.mcis.duke.edu/standards/termcode/loinc.htm.

SNOMED defines code standards in a variety of clinical areas, called coding axes: topography; morphology; function; living organisms; chemicals, drugs, and biologic products; physical agents, activities, and forces; occupations; social context; diseases/diagnoses; procedures; general linkages/modifiers.

### Security and Privacy

Privacy issues include both information technology and policy considerations. For example, security can be addressed by encryption techniques; policies that strongly discourage sharing of passwords are also required for adequate privacy and security.

The public health system has been working to adopt needed standards for immunization data transactions using HL7, data elements for emergency department systems, and an approach for piloting electronic reporting from clinical laboratories (which defines an HL7 message with LOINC codes for identifying tests and SNOMED for identifying results, and a set of tables that define reportable diseases in terms of specific tests and results) (1).

The "rules" for achieving public health goals for electronic clinical data are as follows. 1) Take advantage of the momentum of the existing standards in hospitals and laboratories. 2) Recognize that this is difficult and will take a long time. 3) Consider the source system data structures when defining data needs.

### Opportunities and Pitfalls for Surveillance

William Braithwaite, Department of Health and Human Services, described the Administrative Simplification provision of the Health Insurance Portability and Account Act of 1996 (HIPAA), which is intended to standardize the electronic data interchange of certain administrative and financial transactions while protecting the security and privacy of transmitted information. The act mandates nine transaction standards (e.g., claims, encounters, enrollment) including code sets; coordination of benefits information; unique identifiers (including defining allowed uses) for individuals, employers, health plans, and health-care providers; and security, confidentiality, and electronic signatures. Once standards are adopted, all health plans, clearinghouses, and those providers who choose to conduct transactions electronically will be required to implement them. The time line for implementation calls for adoption by the Secretary of Health and Human Services (HHS) during 1998 of all standards except claim attachments. ("Claim attachments" refers to information requested by an insurance payer from a health-care provider to justify submitted charges and is difficult to standardize because of the diversity of requests.) The Secretary will look first to industry for a consensus standard developed by an ANSI-accredited standards development organization and will rely upon advice of the National Committee on Vital and Health Statistics. The HHS implementation strategy involves a three-tiered approach. 1) The HHS Data Council, a senior level policy guidance and decision-making group, is the contact for the National Committee on Vital and Health Statistics. 2) The Data Council's Health Data Standards Committee provides management of the standards activities. 3) Implementation Teams provide research, analysis, and development of standards and implementing regulations. The HHS adopts a standard by publishing in the Federal Register a Notice of Intent to gather information when no consensus exists and a Notice of Proposed Rule Making, which provides a draft final rule. Publication of a Final Rule marks the "adoption" by HHS of a particular standard.

Standards proposed for adoption include X12N Version 4010 for all transactions except pharmacy claims, for which the National Council for Prescription Drug Program Version 3.2 is proposed. Coding standards proposed for adoption include ICD-9-CM, followed by ICD-10-CM in 2001 for diagnoses, and ICD-9-CM Vol. 3 and Health Care Financing Administration Common Procedure Coding System (HCPCS) for procedures. Proposed identifier standards are the National Provider Identifier Health Care Financing Administration (HCFA) for providers, the PAYERID (HCFA) for health plans; and the Employer Identification Number (Internal Revenue Service) for employers. A Notice of Intent will be published to seek input regarding the individual identifier.

Important issues for public health surveillance in the next phases include participating in development of the data content of these standards, the standard for claim attachments, and the electronic medical records standards, and developing health information privacy that maintains appropriate access to data for public health purposes. Additional information about the Administrative Simplification provisions of HIPAA can be found at http://aspe.os.dhhs.gov/admnsimp/.

### Public Health Surveillance for the 21st Century

Paul Stehr-Green, Washington Department of Health, emphasized public health surveillance as the foundation of public health practice. Public health surveillance needs to adapt to changing health practice, such as requiring assessment of the risks for new and reemerging infectious diseases or environmental hazards.

Public health should use the array of new information tools available. The Blueprint for Surveillance is a document prepared by the Council of State and Territorial Epidemiologists; it outlines the National Public Health Surveillance System. This conceptual framework approaches surveillance for not only reportable diseases, but also for a variety of health outcomes, costs, and risk factors important to public health. The National Public Health Surveillance System would involve other approaches (taking into account available funding levels and the particular goals of surveillance at each level of the public health system) in addition to the traditional reportable diseases surveillance model. The primary goals of the National Public Health Surveillance System include 1) coordinating new and existing public health surveillance systems and linking them to facilitate the exchange of data; 2) encouraging partnerships of federal, state, and local public health professionals in decision-making about surveillance activities; 3) reviewing existing surveillance (and other data collection efforts that have a surveillance component) and making decisions about new surveillance efforts and changes in existing systems; 4) monitoring the adequacy of methods and processes involved in current surveillance systems; and 5) developing a comprehensive description of conditions under surveillance to bring attention to public health surveillance activities and justify the need to support these activities. Recent accomplishments include an effort coordinated by the Centers for Disease Control and Prevention's (CDC) Health Information and Surveillance Systems Board to integrate a number of current surveillance systems; updating the agency's inventory of surveillance systems; developing a policy to monitor and evaluate proposals to develop new or to substantially modify existing surveillance systems; developing an investment analysis policy, which may allow the use of some portion of funds to support the development and maintenance of integrated surveillance and information systems by state health departments; and developing resources that have been made available to state health departments for enhancing infectious diseases surveillance capacity. Washington State is formally reviewing the regulatory foundation for surveillance and is developing and piloting electronic systems for the collection, management, analysis, and dissemination of surveillance data and information, including a collaboration between the health department and Group Health Puget Sound Cooperative to electronically send selected laboratory data to the department of health for surveillance. State and local health departments should commit to changing from old to modern ways of approaching surveillance, and CDC should provide leadership to bring together disparate stakeholders and to provide flexible resources to help state and local health departments effect modernization and integration of surveillance.

### Reference

1. McDonald CF, Overhage JM, Dexter P, Takesue BY, Dwyer DM. A framework for capturing clinical data sets from computerized sources. Ann Intern Med 1997;127:675-82.
